# Association Between Proficiency and Efficiency in Electronic Health Records Among Pediatricians at a Major Academic Health System

**DOI:** 10.3389/fdgth.2021.689646

**Published:** 2021-09-06

**Authors:** Saif Khairat, Lauren Zalla, Allie Gartland, Carl Seashore

**Affiliations:** ^1^Carolina Health Informatics Association, University of North Carolina at Chapel Hill, Chapel Hill, NC, United States; ^2^Cecil G. Sheps Center for HEalth Service Research, University of North Carolina at Chapel Hill, Chapel Hill, NC, United States; ^3^Department of Epidemiology, Gillings School of Global Public Health, University of North Carolina at Chapel Hill, Chapel Hill, NC, United States

**Keywords:** electronic health record, pediatrics, activity, efficiency, proficiency testing

## Abstract

**Objective:** The purpose of this study was to evaluate the variations in electronic health record (EHR) activity among General and Specialty pediatricians by investigating the time spent and documentation length, normalized for workload.

**Materials and Methods:** We conducted a cross-sectional study of pediatric physicians using Epic EHR at a major Southeastern academic healthcare system. We collected user-level EHR activity data of 104 pediatric physicians over 91 days from April 1 to June 30, 2020.

**Results:** Of the 104 pediatrics physicians, 56 (54%) were General pediatricians and 48 (46%) were Specialists pediatricians. General pediatricians spent an average of 17.6 min [interquartile range (IQR): 12.9–37] using the EHR per appointment, while Specialists spent 35.7 min (IQR: 28–48.4) per appointment.

Significant negative associations were found between proficiency scores and the amount of time spent in the system for Generalists (*p* < 0.001). On the contrary, significant positive associations were found between proficiency scores and the amount of time spent in the system for Specialists (*p* < 0.01).

**Conclusions:** We report an association between EHR proficiency and efficiency levels among pediatricians within the same healthcare system, receiving the same EHR training, and using the same EHR system. The profound differences in EHR activity suggest that higher priority should be given to redesigning EHR training methods to accommodate the learning needs of physicians.

## Introduction

Electronic health record (EHR) use is associated with physician burnout and fatigue (1, 2). Burnout is more common among frontline specialties such as family medicine (3), and rates among physicians of all specialties remain above 40% (4). Burnout is associated with more EHR time outside clinic hours, also known as *pajama time* (5), more clinical documentation (6, 7), lower same-day chart completion, longer completion time for inbox messages, and incomplete inbox messages (5–7). Physicians spend nearly half of their total EHR time per day on clerical tasks, documentation, order entry, billing and coding, system security, and administrative tasks and a quarter of their time on managing their in-basket messages (6). More specifically, primary care physicians spend an average of 190 min per day on documentation alone, with documentation being the primary task both in clinic and remotely (7) On average, physicians spend 1–2 h outside of working hours on EHR work (8). Burned-out providers also spend an additional 10 min per appointment after-hours working in the EHR compared with non-burned-out colleagues (2, 3, 9).

There is limited knowledge about the differences in EHR efficiency levels, measured by time and documentation length, among physicians and, particularly, pediatricians (10). We know that three EHR tasks accounted for almost 75% of the time of pediatricians in the EHR: Chart review, documentation, and ordering (such as medication ordering) (11). Additionally, general pediatricians spend more hours on clerical tasks than face-to-face time with patients per day (7). However, the degree of variations in EHR use among pediatricians and the EHR tasks contributing to these variations, which is an important step toward improving overall EHR usability, are unknown. Understanding the degree of variability in EHR use is important to organizations and departments, which examine ways to improve EHR use and to mitigate burnout levels.

EHR usage of providers in primary care is highly variable, even within the same network of clinics that use the same EHR system (12). However, providers who spend more time personalizing their EHR interface tend to be happier and more efficient EHR users (13). For this reason, EHR vendors measure the level of system personalization of providers through composite scores, also known as system proficiency, which is one way to identify areas of improvement and to inform ways to improve EHR efficiency. EHR-generated proficiency scores have been used as a reliable metric to assess evidence-based EHR training (14).

Prior studies investigating the relationship between pediatricians and EHR use were survey-based and subjective (11, 15, 16). Subjective findings report that there is a lack of pediatric-specific EHR functionality, which increases workload and reduces productivity and efficiency (15). Furthermore, no study, to our knowledge, thoroughly examined EHR use patterns among pediatric subspecialties (6). Hence, there is a need for a more objective investigation into how pediatricians use the EHR and the differences in use that may contribute to burnout.

The purpose of this study was to describe the wide variations in EHR use among pediatric physicians by examining the association of system proficiency with efficiency levels, normalized for workload.

## Methods

### Study Design and Participants

We conducted a cohort study of pediatric physicians using Epic EHR (Epic Systems®, Madison, WI) at a major Southeastern academic healthcare system. Using the web-based analytics dashboard of Epic, we collected user-level data on how physicians use the EHR. User-error data was not collected at the time of the study. We assessed the time spent in Epic and documentation length during physician use of chart review, in-basket, and notes. We collected EHR user-level data of all academic pediatric physicians at the University of North Carolina at Chapel Hill Medical Center over 91 days, from April 1to June 30, 2020. Institutional Review Board approval was obtained from the University of North Carolina at Chapel Hill prior to conducting the study.

We limited our analysis to the 104 physicians who practiced pediatric medicine within the system for at least 2 months during the 3-month study period. Because our evaluation was focused on ambulatory practice, we also excluded physicians with unique or highly specialized practices including dentists, anesthesiologists, neurologists, surgeons, hospitalists, and neonatologists, as well as physicians on the child abuse team.

### Outcomes

The primary outcomes examined were average time in the EHR system per appointment and physician proficiency score. The secondary outcomes were average time in completing specific tasks per appointment, average time spent during clinic hours [7A-7P] vs. pajama time [7P-7A], and average total documentation length and progress notes length per appointment.

### Measurements

We collected two types of user-level data relating to the effort: EHR time measured in minutes and EHR documentation measured by characters. EHR time contained five different variables: (1) average time in the system per appointment, (2) average time in chart review per appointment, (3) average time in in-basket per appointment, (4) average time in notes per appointment, (5) average time in the system between 7 p.m. and 7 a.m. (pajama time) per appointment. Time was measured as the time from when the provider logged in into the system and was actively using it, including mouse movement, clicks, scrolling, and keyboard strokes. Time measurements were stopped when there was a 5-s inactivity timeout or when the provider logged out of the system. EHR documentation contained two different variables: (1) average documentation length per appointment, (2) average progress notes length per appointment.

Each provider received a proficiency score at the end of each monthly report. Proficiency scores are designed as composite scores from 0 to 10 aimed to measure the personalization and utilization level of the provider. Epic has built-in tools that are intended to improve user efficiency. Proficiency scores, generated by the EHR system, are defined as how frequently the provider used the following efficiency tools: Chart search, SmartTools, QuickAction, and speed buttons. The following scaling system was used to calculate the monthly proficiency score for each participating physician:

- points per QuickAction used, up to 100 uses
- points per provider preference list entry, up to 100 entries
- 1.2 points per 10% of notes written using SmartTools
- points for having a customized level of service speed buttons
- points for having customized diagnosis speed buttons
- points for using Chart search.

### Data Analysis

We calculated descriptive and summary statistics for the primary and secondary outcomes, stratified by physician type (Generalist vs. Specialist). EHR use data were collected by the provider at the end of each month. To standardize measures of EHR use over time and across physicians with different workloads, we first summed each outcome over the 2- or 3- month observation period and then divided it by the total number of appointments of the physician during the observation period.

We computed descriptive statistics stratified by physician type (Generalist vs. Specialist). Then, within each group, we further examined the differences between most efficient and least efficient EHR users. High-performing users were the top 10% of physicians who spent the least amount of time in the EHR per appointment. Low-performing users were the bottom 10% of physicians who spent the most amount of time in the EHR per appointment. We computed Pearson's correlation coefficients to examine the strength of the linear association between physician proficiency score and time spent in the EHR. All analyses were performed in SAS (Version 9.4, Cary, NC).

## Results

Of the 104 pediatrics physicians, 56 (54%) were Generalists and 48 (46%) were pediatric Specialists, including 13 hematologists and oncologists (27.1%), 6 pulmonologists (12.5%), 5 cardiologists (10.4%), 5 geneticists (10.4%), 4 endocrinologists (8.3%), 4 gastroenterologists (8.3%), 4 allergists and immunologists (8.3%), 3 nephrologists (6.3%), 3 infectious disease specialists (6.3%), and 1 rheumatologist (2.1%).

General pediatricians spent an average of 17.6 min (IQR: 12.9–37) using the EHR per appointment, while Specialists spent 35.7 min (IQR: 28–48.4) per appointment (Table 1).

**Table 1:** Distribution of electronic health records activity adjusted per appointment for General and Specialty pediatricians between April 1 and June 30, 2020.

	**General pediatrics (*****n*** **= 56)**					**Specialty pediatrics (*****n*** **= 48)**				
	**Minimum**	**25th Percentile**	**Median**	**75th Percentile**	**Maximum**	**Minimum**	**25th Percentile**	**Median**	**75th Percentile**	**Maximum**
Average documentation length per appt (chars)	1,022	5,725	7,019	9,147	18,861	735	7,881	10,806	14,042	23,935
Average progress note length per appt (chars)	535	3,317	4,003	4,648	7,569	135	4,556	6,244	7,215	12,313
Average time in system per appt (Min)	2.4	12.9	17.6	37.0	141.3	9.4	28.0	35.7	48.4	93.7
Average time in chart review per appt (Min)	0.4	1.6	2.1	5.2	51.4	2.3	4.2	5.3	8.4	14.4
Average time in basket per appt (Min)	0.2	1.1	1.9	3.4	14.3	0.7	3.9	5.3	8.2	21.4
Average time in notes per appt (Min)	0.6	4.3	6.7	11.4	35.6	1.8	7.8	12.9	17.7	37.1
Average time outside of 7AM-7PM per appt (Min)	0.0	0.0	0.3	1.1	7.4	0.0	0.1	0.8	2.8	13.3
Average proficiency score (0-10)	1.7	4.9	6.6	6.8	8.9	0.9	2.8	5.3	5.9	7.9

*Appt, appointment; chars, characters; min, minutes*.

Pediatricians spent the most amount of time in the EHR in Notes for both Generalists (Median: 6.7 min; IQR: 4.3–11.4) and Specialists (Median: 12.9 min; IQR: 7.8–17.7). While the least amount of time was spent in the system when outside of working hours for Generalists (Median: 0.3 min; IQR: 0–1.1) and Specialists (Median: 0.8 min; IQR: 0.1–2.8).

**Figure 1:**
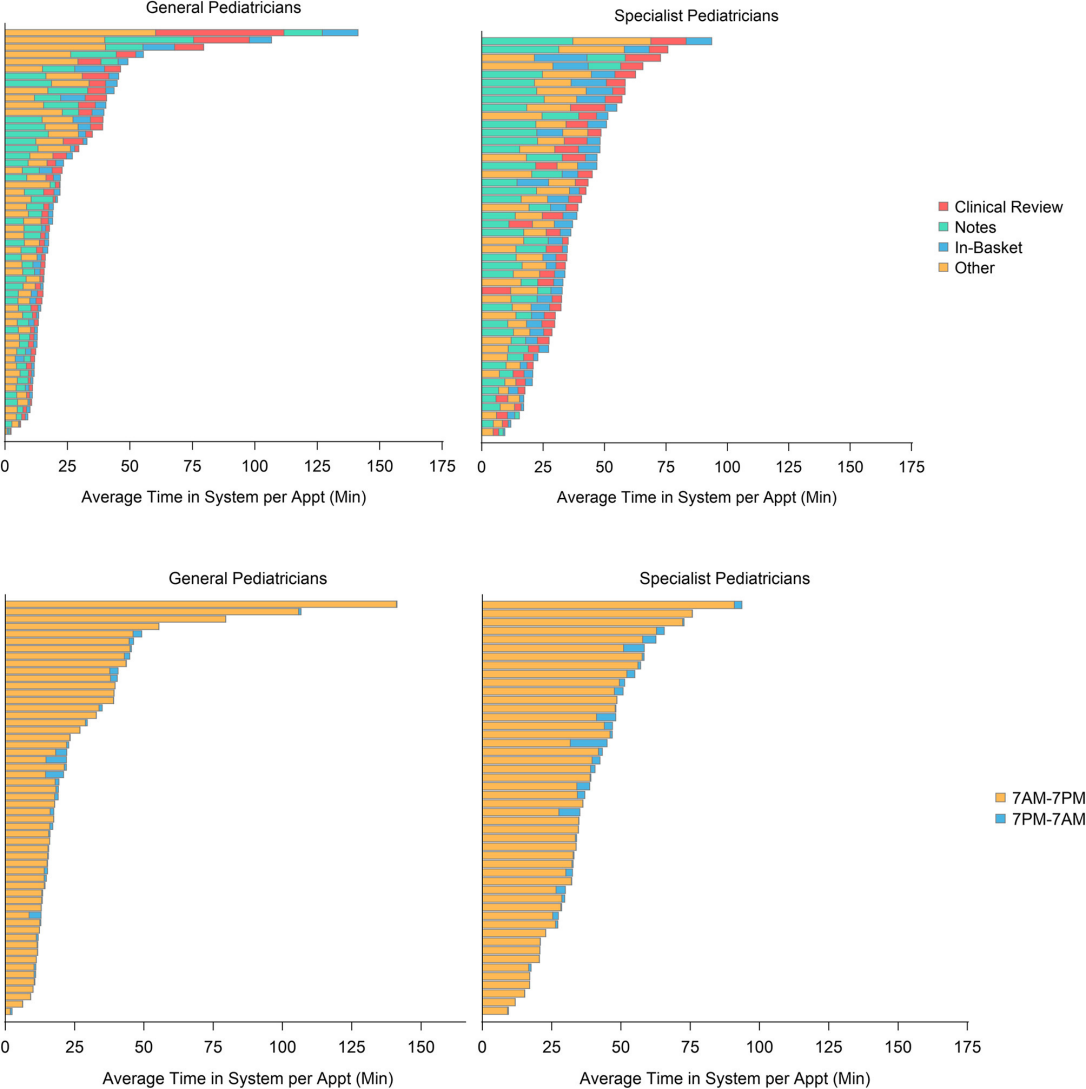
Average amount of time spent in the electronic health record (EHR) system per appointment, classified by time of day and type of activity, among 56 General pediatricians and 48 Specialist pediatricians between April 1 and June 30, 2020.

Among both Generalists and Specialists, there were substantial variations in the amount of time spent in the EHR system and in EHR tasks completed, both overall and by appointment (Figure 1). Among Generalists, the average amount of time spent in the system per appointment ranged from 2.4 to 141 min. Among Specialists, the average amount of time in the system per appointment ranged from 9.4 to 93 min.

**Figure 2:**
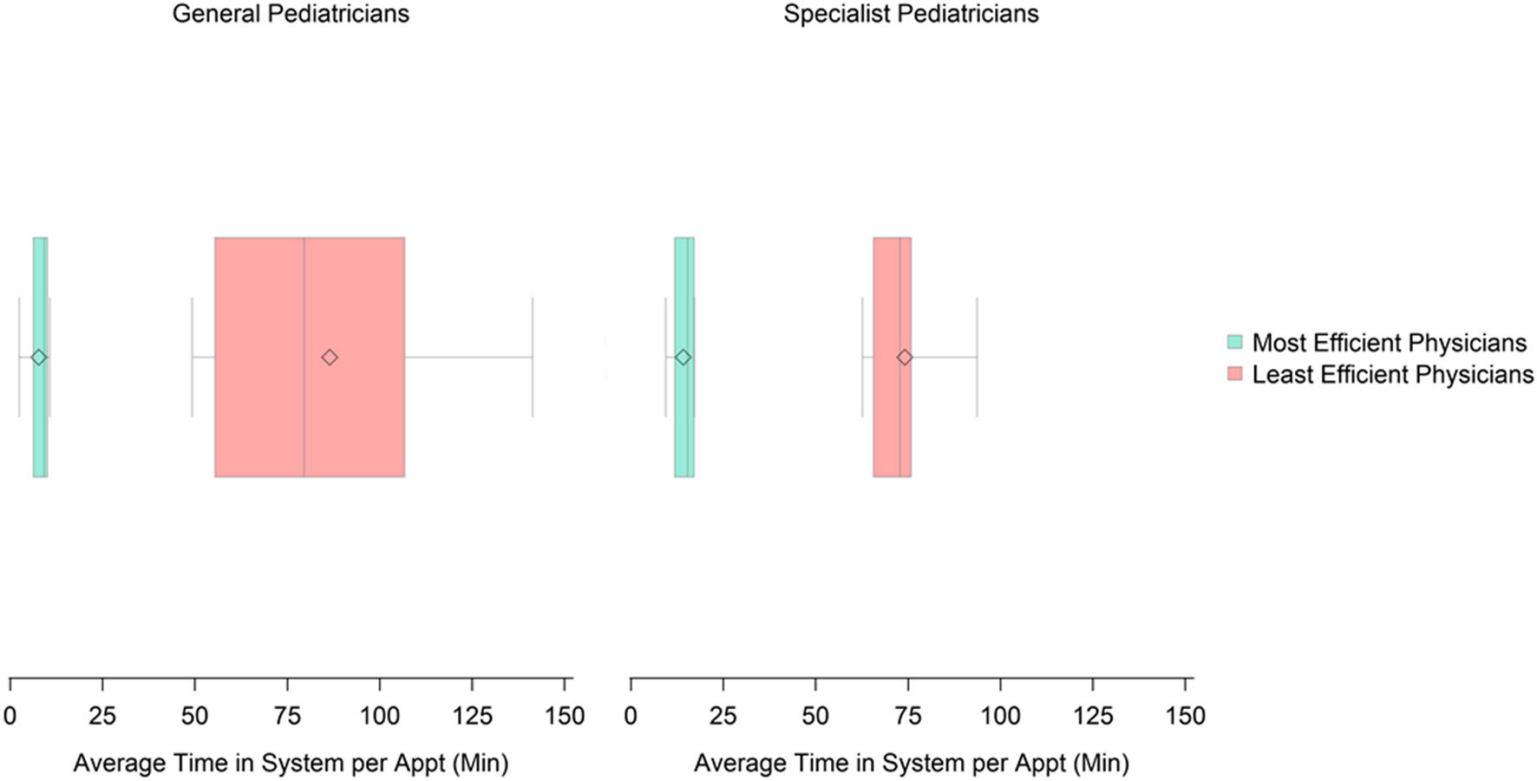
Distribution of time spent in the EHR system per appointment among the most and least efficient General and Specialist pediatricians.

Among Generalists, the average amount of time spent using the EHR per appointment for the most efficient users was 9.1 min (range: 2.4–10.7) compared to 79.5 min (range: 49.2–141.3) for the least efficient users (Figure 2). Among Specialists, the average amount of time spent using the EHR per appointment for the most efficient users was 15.3 min (range: 9.4–17.1) compared to 72.8 min (range: 62.6–93.7) for the least efficient users.

### Electronic Health Record Proficiency

The average EHR proficiency score for Generalists was 6.6 (IQR: 4.9–6.8) and for Specialists was 5.3 (IQR: 2.8–5.9). Wide variations in proficiency scores were found for both Generalists (1.7–8.9) and Specialists (0.9–7.9).

Significant negative associations were found between proficiency scores and the amount of time spent in the system by Generalists (r = −0.43; *p* = 0.009). More proficient Generalists spent less time in the EHR (Table 2). On the contrary, a significant positive association was found between proficiency scores and the amount of time spent in the system by Specialists (r = 0.39; *p* = 0.007), Specialists with higher proficiency scores spent more time in the system. A marginal positive association was found between proficiency scores and documentation length for Specialists (r = 0.26; *p* = 0.07).

**Table 2:** Pearson correlation of EHR proficiency score with time in system and documentation length for 56 General pediatricians and 48 Specialist pediatricians between April 1 and June 30, 2020.

**Proficiency score**	**Average time in the system per appointment**	**Average documentation length per appointment**
Generalist	−0.34 (*p* = 0.009)^*^	−0.14 (*p* = 0.317)
Specialist	0.39 (*p* = 0.007)^*^	0.26 (*p* = 0.075)^**^

* *Denotes statistical significance at p < 0.05*.

** *Denotes marginal significance at p < 0.1*.

## Discussion

This study demonstrates that a wide gap in EHR usability exists between pediatricians within the same healthcare system, receiving the same EHR training, and using the same EHR system. We report substantial variations in EHR screen time and documentation length among pediatricians after adjusting to the workload. We found that time spent and documentation length in progress notes were the highest contributors to the observed, which can provide insights into the effect of suboptimal EHR use on physician burnout.

EHR proficiency was significantly associated with time spent in the system. Generalist pediatricians typically practice in primary care clinics where the type of care provided is usually repetitious and direct, which explains why higher EHR proficiency was associated with less time in the system. On the contrary, higher EHR proficiency was associated with more time in the system among Specialists. This may be because Specialists see more complex patients and, therefore, need more time to review the patient chart and to respond to in-basket messages. These results raise the question of whether more refinements are needed to improve the usability and integration of efficiency tools within Specialty practice. This suggests that EHR proficiency levels may be associated with the quality of patient care (17).

Prolonged time of using the EHR is associated with reduced physician efficiency and increased fatigue levels (2). EHR inefficiencies are attributed to human factors and EHR interface design issues (18, 19). Human factors that contribute to inefficient use of EHRs include provider demographics such that women were found to be more efficient users of the EHR because of their use of search functions and filters to find patient information in the EHR (9). The professional role also explains efficiency variations where more senior clinicians were less efficient EHR users using more clicks and spending more time to complete a task when navigating the EHR compared to younger trainees (18, 20, 21). EHR interface design issues that lead to inefficiency include the overuse of menus, the lack of data visualization, and the lack of customization (22, 23).

Improving EHR training has been associated with increased EHR efficiency levels and improved physician well-being (24, 25). While this study showed wide variations in EHR efficiency among pediatricians, one way to bridge the disparities in EHR efficiency is to optimize the traditional EHR training courses that may lack efficacy due to the didactic structure and instructor-led design without being physician-centric. Physicians, like medical students, are divided into four types of learners: visual, auditory, reading/writing preference, and kinesthetic.

The substantial differences in EHR documentation could be linked to best practices conveyed during training or through peer-to-peer assistance. Stringent documentation policies may play a role in over-documentation of some physicians, which warrants further investigation to determine if the short documentation of the most efficient EHR users is comparable in quality and meet policy requirements as the longer documentation of the least efficient users.

### Limitations and Future Direction

Although we adjusted our analysis by appointment, the type of appointment between new and current patients and the complexity of patients was not collected, which may contribute to the differences in EHR activity. We realize that, while there have been limitations reported to the use of some EHR logs, Epic conducted multiple improvements on their audit log data reporting; nevertheless, we could not validate the EHR usage data we obtained (26). Additionally, proficiency scores were automatically computed by the EHR vendor and could not be further validated. However, this study provides real-time information on the differences in EHR use among pediatrics physicians, which may suggest tailoring EHR training techniques to improve physician use of the EHR and physician well-being. The healthcare system switched all unnecessary in-person visits to virtual visits during March 2020 due to the pandemic and gradually resumed in-office visits shortly thereafter. Our hypothesis was that any workflow changes will affect all providers equally although physicians were still required to do pre-visit planning and post-visit documentation. Nonetheless, one limitation of this study is that we did not account for the transformation to virtual visits and its effect on EHR use.

In the future, the integration of Keystroke Level Model (KLM) data can help validate EHR audit data as well as provide further insights into the estimated task execution time for providers, which can then be compared against audit data to identify high-performing and low-performing users.

## Conclusions

Poor EHR use is a major contributor to physician burnout. In this study, we report profound differences in EHR use, measured by time and effort, among physicians when adjusted for workload. These findings suggest that the current EHR training framework does not meet the different learning styles, which means more thorough changes to current EHR training sessions are needed to improve the usability of EHRs and the well-being of physicians.

## Data Availability Statement

The datasets presented in this article are not readily available because data includes provider level data, including identifiable data, on EHR use. Requests to access the datasets should be directed to Saif Khairat, saif@unc.edu.

## Ethics Statement

The studies involving human participants were reviewed and approved by University of North Carolina at Chapel Hill. Written informed consent for participation was not required for this study in accordance with the national legislation and the institutional requirements.

## Author Contributions

SK contributed to conception and design of the work, to analysis or interpretation of data for the work, and to manuscript writing. LZ contributed to data analysis or interpretation of data for the work and to manuscript writing. AG contributed to manuscript writing. CS contributed to the conception and design of the work, to data collection, to analysis of the data, and to manuscript writing. All authors contributed to the article and approved the submitted version.

## Conflict of Interest

The authors declare that the research was conducted in the absence of any commercial or financial relationships that could be construed as a potential conflict of interest.

## Publisher's Note

All claims expressed in this article are solely those of the authors and do not necessarily represent those of their affiliated organizations, or those of the publisher, the editors and the reviewers. Any product that may be evaluated in this article, or claim that may be made by its manufacturer, is not guaranteed or endorsed by the publisher.
